# Supplementary material for: Influence of different types of sessile epibionts on the community structure of mobile invertebrates in an eelgrass bed
Source: PeerJ. 2017 Jan 31;5:e2952. doi: 10.7717/peerj.2952 (PMC5289102; doi:10.7717/peerj.2952)
Supplement: Table S1 — The biomass proportion and the rank are also shown. [file peerj-05-2952-s001.docx]

**Supplemental Table**

|  | | | | | | | | | | |
| --- | --- | --- | --- | --- | --- | --- | --- | --- | --- | --- |
| **Table S1.** Biomass of 32 mobile invertebrates at seven stations in the Akkeshi-ko estuary and Akkeshi Bay. The biomass proportion and the rank are also shown. | | | | | | | | | | |
| **Species** | **Mean AFDW ± SD (mg m^-2^: n=3 per site)** | | | | | | | | **Prop. (%)** | **Rank** |
|  | **BK** | **HN** | **TB** | **SL** | **CL** | **CK** | **SR** | **Among sites** |  |  |
| **Gammarid amphipoda** | | | | | | | | | | |
| *Ampithoe lacertosa* | 2908 ± 745 | 1283 ± 1201 | 566 ± 510 | 71 ± 52 | 1733 ± 705 | 1183 ± 1105 | 31 ± 34 | 1107 ± 1156 | 17.97 | 3 |
| *Aoroides curvipes* | 15 ± 26 | ― | 43 ± 22 | ― | 2 ± 4 | ― | ― | 9 ± 19 | 0.14 | 21 |
| *Ischyroceridae* sp. | ― | ― | ― | ― | ― | ― | 24 ± 25 | 3 ± 12 | 0.06 | 26 |
| *Grandidierella japonica* | ― | ― | ― | ― | 54 ± 94 | 15 ± 26 | ― | 10 ± 37 | 0.16 | 20 |
| *Hyalidae* sp. | ― | ― | ― | ― | ― | ― | 39 ± 68 | 6 ± 26 | 0.09 | 23 |
| *Monocorophium* spp. | 1131 ± 251 | 1 ± 2 | 3 ± 5 | 1 ± 1 | 49 ± 16 | 24 ± 23 | 2 ± 4 | 173 ± 409 | 2.81 | 8 |
| *Pleustes panoplus* | ― | ― | ― | ― | 6 ± 11 | ― | ― | 1 ± 4 | 0.01 | 28 |
| *Pontogeneia rostrata* | 21 ± 16 | 256 ± 139 | 6 ± 11 | 73 ± 25 | 237 ± 166 | 146 ± 161 | 1 ± 2 | 106 ± 134 | 1.72 | 12 |
| **Caprellid amphipoda** | | | | | | | | | | |
| *Caprella acanthogaster* | ― | ― | ― | ― | 152 ± 152 | ― | ― | 22 ± 73 | 0.22 | 18 |
| *Caprella kroyeri* | 58 ± 100 | 99 ± 126 | 135 ± 155 | 10 ± 15 | ― | ― | ― | 43 ± 88 | 0.61 | 16 |
| *Caprella laeviuscula* | 403 ± 349 | ― | ― | ― | 132 ± 229 | ― | 5 ± 8 | 77 ± 195 | 0.79 | 13 |
| *Caprella scaura* | ― | ― | ― | ― | ― | 44 ± 76 | ― | 6 ± 29 | 0.06 | 25 |
| *Caprella tsugarensis* | ― | ― | ― | 30 ± 51 | 35 ± 40 | 286 ± 294 | 9 ± 16 | 51 ± 137 | 0.52 | 17 |
| **Isopoda** | | | | | | | | | | |
| *Cymodoce japonica* | 16 ± 28 | 221 ± 324 | 79 ± 68 | 12 ± 19 | 754 ± 1300 | 870 ± 779 | ― | 279 ± 605 | 4.47 | 5 |
| *Munna* sp. | ― | 1 ± 1 | ― | 23 ± 17 | 2 ± 1 | 6 ± 7 | ― | 4 ± 10 | 0.07 | 24 |
| *Paranthura japonica* | ― | 157 ± 147 | 604 ± 319 | 39 ± 68 | 306 ± 271 | 734 ± 469 | ― | 263 ± 351 | 4.27 | 7 |

| **Table S1.** (continued) | | | | | | | | | | |
| --- | --- | --- | --- | --- | --- | --- | --- | --- | --- | --- |
| **Species** | **Mean AFDW ± SD (mg m^-2^: n=3 per site)** | | | | | | | | **Prop. (%)** | **Rank** |
|  | **BK** | **HN** | **TB** | **SL** | **CL** | **CK** | **SR** | **Among sites** |  |  |
| **Copepoda** |  |  |  |  |  |  |  |  |  |  |
| *Kushia zosteraphila* | 1 ± 1 | ― | 54 ± 47 | 37 ± 19 | 2 ± 2 | 13 ± 14 | 18 ± 7 | 11 ± 16 | 0.18 | 19 |
| Miraciidae sp. | ― | 1 ± 1 | ― | ― | 4 ± 5 | ― | ― | 1 ± 4 | 0.01 | 29 |
| Thalestridae sp. | ― | ― | ― | ― | 3 ± 2 | ― | ― | 1 ± 1 | 0.01 | 30 |
| **Cumacea** | | | | | | | | | | |
| *Diastylis* sp. | ― | ― | ― | ― | 2 ± 4 | ― | ― | 0 ± 2 | 0.01 | 31 |
| **Gastropoda** |  |  |  |  |  |  |  |  |  |  |
| *Ansola angustata* | 1187 ± 819 | ― | ― | ― | ― | ― | ― | 170 ± 498 | 2.78 | 9 |
| *Batillaria attramentaria* | ― | ― | ― | 314 ± 544 | ― | ― | ― | 45 ± 205 | 0.73 | 15 |
| *Lacuna smithi* | 1 ± 1 | ― | ― | ― | ― | ― | ― | 0 ± 0 | 0.00 | 32 |
| *Lacuna* spp. | 318 ± 205 | 3 ± 4 | 1 ± 1 | ― | 162 ± 166 | 2342 ± 2267 | 7164 ± 2147 | 1427 ± 2717 | 23.42 | 2 |
| *Nassarius fraterculus* | ― | ― | ― | ― | 9 ± 15 | ― | ― | 1 ± 6 | 0.02 | 27 |
| *Siphonacmea oblongata* | ― | ― | ― | ― | 17 ± 30 | ― | 1838 ± 953 | 265 ± 724 | 4.36 | 6 |
| **Polychaeta** | | | | | | | | | | |
| *Exogone naidina* | ― | ― | ― | ― | 45 ± 31 | 1995 ± 2141 | ― | 291 ± 983 | 4.57 | 4 |
| *Harmothoe imbricata* | ― | ― | ― | ― | 310 ± 248 | 35 ± 60 | ― | 49 ± 136 | 0.77 | 14 |
| *Nereis* sp. | ― | ― | ― | ― | 2333 ± 2873 | 8741 ± 6504 | ― | 1582 ± 3835 | 24.62 | 1 |
| *Syllis* sp. | ― | ― | 585 ± 1013 | 208 ± 208 | 181 ± 262 | 14 ± 25 | ― | 141 ± 395 | 2.22 | 11 |
| **Platyhelminthes** | | | | | | | | | | |
| Rhabdocoela sp. | 4 ± 5 | 10 ± 3 | 20 ± 19 | 17 ± 8 | ― | 2 ± 3 | ― | 8 ± 10 | 0.12 | 22 |
| **Hirudinoidea** | | | | | | | | | | |
| *Ostreobdella kakibir* | ― | ― | ― | ― | ― | ― | 1097 ± 1552 | 157 ± 629 | 2.22 | 10 |
